# Multi-ancestry, trans-generational GWAS meta-analysis of gestational diabetes and glycaemic traits during pregnancy reveals limited evidence of pregnancy-specific genetic effects

**DOI:** 10.1038/s41467-026-73509-y

**Published:** 2026-06-01

**Authors:** Caroline Brito Nunes, Valentina Rukins, Aminata H. Cisse, Frédérique White, Nancy McBride, Alan Kuang, Catherine Allard, Justiina Ronkainen, Alice Hughes, Amanda Elliott, Gudmar Thorleifsson, Marc Vaudel, Triin Laisk, Yuqin Gu, Amel Lamri, Li Chen, Johanna Tuhkanen, Jari Lahti, Lucinda Calas, Manon Muntaner, Ville Karhunen, Jaewon Choi, Anni Heiskala, Gad Hatem, Nicolas Fragoso-Bargas, Sheryl L. Rifas-Shiman, Guillermo Paz-Lopez, Sara E. Stinson, Bishwajit Bhowmik, Emma Ahlqvist, “Estonian Biobank Research Team”, Reedik Mägi, Valentina Rukins, “Genes & Health Research Team”, Sarah Finer, Julia Zöllner, Jean-Francois Deleuze, Johan G. Eriksson, Jongseok Park, Koon Teo, Katri Räikkönen, Kari Stefansson, Maria Molina-Vega, Padmaja Subbarao, Robin N. Beaumont, Stefan Johansson, Tiinamaija Tuomi, Torben Hansen, Line Engelbrechtsen, Sonsoles Morcillo, Emily Oken, Elisabeth Quigstad, Kåre I. Birkeland, Marja Vääräsmäki, Andrew T. Hattersley, Sylvain Sebert, Graham A Hitman, Soo Heon Kwak, Marjo-Riitta Järvelin, Rashmi B Prasad, Barbara Heude, Aline Meirhaeghe, Luigi Bouchard, Pierre-Étienne Jacques, Hannele Laivuori, Kok Hian Tan, Sonia S. Anand, David A. van Heel, Siyang Liu, Pål R. Njølstad, Valgerdur Steinthorsdottir, Elisabeth Widén, Elina Keikkala, Denise M Scholtens, William L. Lowe, Sarah Finer, Andrew P Morris, Reedik Mägi, Julia Zöllner, Maria Carolina Borges, Deborah A. Lawlor, Marie-France Hivert, Rachel M. Freathy, David M Evans, Gunn-Helen Moen

**Affiliations:** 1 Institute for Molecular Bioscience, The University of Queensland, St. Lucia, Queensland, Australia; 2 Estonian Genome Centre, Institute of Genomics, University of Tartu, Tartu, Estonia; 3 Department of Clinical and Biomedical Sciences, Faculty of Health and Life Sciences, University of Exeter, Exeter, UK; 4 Département de Biologie, Faculté des Sciences, Université de Sherbrooke, Sherbrooke, Québec, Canada; 5 MRC Integrative Epidemiology Unit at the University of Bristol, UK; 6 Population Health Science, Bristol Medical School, University of Bristol, UK; 7 Department of Preventive Medicine, Northwestern University Feinberg School of Medicine, Chicago, USA; 8 Centre de recherche du Centre Hospitalier Universitaire de Sherbrooke, Sherbrooke, Québec, Canada; 9 Research Unit of Population Health, Faculty of Medicine, University of Oulu, Oulu, Finland; 10 Analytic and Translational Genetics Unit, Massachusetts General Hospital, Boston, MA, USA; 11 Stanley Center for Psychiatric Research, Broad Institute of Harvard and MIT, Cambridge, MA, USA; 12 Harvard Medical School, Boston, MA, USA; 13 deCODE genetics/Amgen, Inc., 102 Reykjavik, Iceland; 14 Mohn Center for Diabetes Precision Medicine, Department of Clinical Science, University of Bergen, Bergen, Norway; 15 Department of Genetics and Bioinformatics, Norwegian Institute of Public Health, Bergen, Norway; 16 School of Public Health (Shenzhen), Shenzhen Campus of Sun Yat-sen University, Shenzhen, China; 17 Department of Medicine, McMaster University, Hamilton, Ontario, Canada; 18 Population Health Research Institute, Hamilton Health Sciences, Hamilton, Ontario, Canada; 19 Institute for Human Development and Potential, Agency for Science, Technology and Research (A*STAR), Singapore, Singapore; 20 Department of Psychology, Faculty of Medicine, University of Helsinki, Finland; 21 Folkhälsan Research Centre, Helsinki, Finland; 22 Université Paris Cité and Université Sorbonne Paris Nord, Inserm, INRAE, Center for Research in Epidemiology and StatisticS (CRESS), Paris, France; 23 Université de Lille, Inserm, CHU Lille, Institut Pasteur de Lille, U1167 – RID-AGE – Facteurs de risque et déterminants moléculaires des maladies liées au vieillissement, Lille, France; 24 Department of Biomedical Sciences, Seoul National University College of Medicine, Seoul, Republic of Korea; 25 Research Unit of Population Health, Faculty of Medicine, University of Oulu, Oulu, Finland; 26 Department of Clinical Sciences, Genetics and Diabetes, Lund University, Malmö, Sweden; 27 Institute of Clinical Medicine, Faculty of Medicine, University of Oslo, Oslo, Norway; 28 Department of Population Medicine, Harvard Medical School and Harvard Pilgrim Health Care Institute, Boston, MA, USA; 29 Unidad de Bioinformática, Instituto de Investigación Biomédica de Málaga y Plataforma en Nanomedicina - IBIMA Plataforma BIONAND, Málaga, Spain; 30 Novo Nordisk Foundation Center for Basic Metabolic Research, University of Copenhagen, Copenhagen, Denmark; 31 Section for Precision Psychiatry, Division of Mental Health and Addiction, Oslo University Hospital, Oslo, Norway; 32 Centre for Global Health Research, Diabetic Association of Bangladesh, Dhaka, Bangladesh; 33 Centre National de Recherche en Génomique Humaine (CNRGH), Université Paris-Saclay, CEA, Evry, France; 34 Department of Obstetrics and Gynaecology and Human Potential Translational Research Programme, Yong Loo Lin School of Medicine, National University of Singapore, Singapore; 35 Department of General Practice and Primary Health Care, University of Helsinki, Helsinki, Finland; 36 Faculty of Medicine, University of Iceland, 101 Reykjavik, Iceland; 37 Unidad de Gestión Clínica Endocrinología y Nutrición, Hospital Universitario Virgen de la Victoria, Málaga, Spain; 38 Instituto de Investigación Biomédica de Málaga y Plataforma en Nanomedicina- IBIMA Plataforma BIONAND, Málaga, Spain; 39 Centro de Investigación Biomédica en Red de la Fisiopatología de la Obesidad y la Nutrición (CIBERObn), Instituto de Salud Carlos III, Madrid, Spain; 40 Hospital for Sick Children (SickKids), Department of Pediatrics, University of Toronto, Toronto, Canada; 41 Dalla Lana School of Public Health, University of Toronto, Toronto, Canada; 42 Department of Medical Genetics, Haukeland University Hospital, Bergen, Norway; 43 Institute for Molecular Medicine Finland (FIMM), Helsinki Institute of Life Science, University of Helsinki, Helsinki, Finland; 44 Department of Endocrinology, Abdominal Centre, Helsinki University Hospital, Helsinki, Finland; 45 Department of Gynaecology and Obstetrics, Herlev Hospital, University of Copenhagen, Herlev, Denmark; 46 Department of Clinical Medicine, Faculty of Health, University of Copenhagen; 47 Department of Endocrinology, Morbid Obesity and Preventive Medicine, Oslo University Hospital, Oslo, Norway; 48 Research Unit of Clinical Medicine, Medical Research Center, Oulu University Hospital, University of Oulu, Oulu, Finland; 49 Finnish Institute for Health and Welfare, Department of Public Health, Welfare Epidemiology and Monitoring Unit, Helsinki and Oulu, Finland; 50 Centre for Genomics and Child Health, Blizard Institute, Faculty of Medicine and Dentistry, Queen Mary University of London, London, UK; 51 Department of Internal Medicine, Seoul National University Hospital, Seoul, Republic of Korea; 52 School of Public Health, Dept of Epidemiology and Biostatistics, Imperial College London, St Mary’s Hospital, London, UK; 53 Department of Biochemistry and Functional Genomics, Faculty of Medicine and Health Sciences, Université de Sherbrooke, Sherbrooke, Québec, Canada; 54 Clinical Department of Laboratory Medicine, Centre intégré universitaire de santé et de services sociaux (CIUSSS) du Saguenay–Lac-St-Jean, Québec, Canada; 55 Center for Child, Adolescent and Maternal Health Research, Faculty of Medicine and Health Technology, Tampere University, Tampere, Finland; 56 Department of Obstetrics and Gynecology, Tampere University Hospital, The Wellbeing Services County of Pirkanmaa, Tampere, Finland; 57 Medical and Clinical Genetics, University of Helsinki and Helsinki University Hospital, Helsinki, Finland; 58 KK Women’s and Children’s Hospital, Singapore, Singapore; 59 Duke-NUS Medical School, Singapore, Singapore; 60 Blizard Institute, Queen Mary University of London; 61 Department of Pediatrics and Adolescents, Haukeland University Hospital, Bergen, Norway; 62 Department of Medicine, Northwestern University Feinberg School of Medicine, Chicago, USA; 63 Wolfson Institute of Population Health, Queen Mary University of London; 64 Centre for Musculoskeletal Research, The University of Manchester, Manchester, UK; 65 NIHR Manchester Biomedical Research Centre, Manchester University NHS Foundation Trust, Manchester Academic Health Science Centre, Manchester, United Kingdom; 66 Women’s Health, University College London, UK; 67 Diabetes Unit, Massachusetts General Hospital, Boston, USA; 68 Departement de Medicine, Faculty of Medicine and Health Sciences, Universite de Sherbrooke, Quebec, Canada; 69 University of Queensland Frazer Institute, University of Queensland, Woolloongabba, Australia

## Abstract

Gestational diabetes mellitus (GDM) affects ~14% of pregnancies and increases maternal type 2 diabetes mellitus (T2DM) risk. The GenDiP Consortium presents trans-generational, multi-ancestry genome-wide association study meta-analyses of GDM and pregnancy glycemic traits in up to 38,305 GDM cases and 776,145 controls. We identify 37 GDM-associated loci (7 novel) and five novel loci for pregnancy glycemic traits, all operating through the maternal genome. We classify 12 GDM variants with stronger effects on GDM than T2DM into five biologically informed categories, revealing pleiotropy patterns, pregnancy-dependent effect modification, and diagnostic heterogeneity. While all these loci overlap with T2DM and/or non-pregnant glycaemic traits, four (*G6PC2, CAST-PCSK1, HKDC1, FOXA2*) lack genome-wide-significant T2DM associations; *GCK* shows distinct causal variants for GDM, and *MTNR1B* exhibits pregnancy-amplified effects. Our findings provide new genetic insights into GDM and highlight the need for larger, ancestrally diverse studies of GDM and glycaemic traits during pregnancy to understand potential pregnancy-specific effects.

Gestational diabetes mellitus (GDM) affects ~14.0% of pregnancies worldwide, and poses risks to both maternal and fetal health^1,2^. Prevalence varies across regions, with the highest rates in Middle Eastern and North African countries (27.6%) and the lowest in North America and the Caribbean (7.1%). This variation is influenced by population characteristics (e.g. ancestry and obesity rates), and differences in screening strategies and diagnostic criteria^3^.

Pregnancy induces metabolic changes essential for fetal development, including increased insulin resistance, heightened insulin secretion, and fluctuations in glucose levels^4–10^. While fasting glucose (FG) levels typically decline in the first trimester before stabilising, postprandial glucose levels tend to rise due to insulin resistance^4,8,10^. GDM arises when these adaptations are disrupted - either by excessive insulin resistance and/or insufficient insulin secretion - leading to maternal hyperglycemia^5,11,12^. While maternal glucose crosses the placenta, maternal insulin does not. In response, elevated maternal glucose levels trigger fetal insulin secretion, contributing to fetal overgrowth and potentially adverse outcomes such as obstructed labour, preterm delivery, operative delivery, stillbirth and neonatal hypoglycaemia^13^.

A previous diagnosis of GDM carries an 8-10-fold higher risk of type 2 diabetes mellitus (T2DM)^13–17^. GDM and T2DM share genetic and lifestyle risk factors^18–22^, with recent genome-wide association studies (GWASs) reporting a strong genetic correlation between the two conditions (rG ~ 0.70), and only a few genome-wide significant loci unique to GDM^21,23^. However, while large-scale multi-ancestry GWAS of T2DM have identified hundreds of loci and included millions of individuals^24–26^, GDM GWAS have been small in comparison^21,23,27–31^. The disparity in sample size between GDM and T2DM meta-analyses limits attempts to understand whether GDM and T2DM represent the same condition (i.e. the physiological stress of pregnancy unmasking a predisposition to T2DM) or the extent to which there are distinct genetic determinants of both.

The largest and most recent GDM GWASs to date include the FinnGen study (12,332 cases; 131,109 controls; European ancestry)^23^ and the MONN study (12,024 cases; 67,845 controls; East Asian ancestry)^31^, which together identified 27 loci associated with GDM. Both these large GWASs, however, were limited to single ancestral populations, unlike the previous Genetics of Diabetes in Pregnancy (GenDiP) GWAS (5,485 cases, 347,856 controls)^21^ and this current GWAS.

Further insights into the genetic aetiology of GDM may come from GWAS of glycemic traits during pregnancy, such as FG, 1-hour (1hG) and 2-hour (2hG) post-oral glucose tolerance test (OGTT) glucose levels and glycated haemoglobin (HbA1c)^30–35^. These traits capture variation in maternal glucose regulation and may reveal loci influencing glycemic physiology not detectable through binary GDM analyses. A recent GWAS in East Asian mothers (up to 85,856 individuals) identified distinct genetic architectures for fasting versus post-OGTT glucose regulation, suggesting pregnancy-specific effects at several loci^30,31^. However, this study was limited to East Asians, and critical gaps remain in determining whether glycemic trait regulation during pregnancy differs across populations.

In addition to maternal genetic contributions, recent studies have highlighted the role of fetal genetics in shaping the prenatal environment independently of the maternal genome. While it is expected that fetal genetic variants influence fetal traits such as birth weight and placental weight, emerging evidence suggests that the fetal genome may also influence maternal conditions, such as pre-eclampsia, gestational hypertension, and gestational duration - even after accounting for maternal genetics^36–43^. Studies also support a potential role of fetal imprinted genes in modulating maternal insulin resistance and fasting responses via placental hormone signalling^44,45^. However, studies often fail to account for the correlation between maternal and fetal genotypes, limiting conclusions about independent fetal effects on GDM.

In this work, we perform the largest multi-ancestry and trans-generational GWAS meta-analyses of GDM and glycemic traits during pregnancy to date (Figure 1). We perform downstream analyses with our two largest ancestry groups - Europeans and East Asians - to elucidate the degree to which GDM and T2DM represent the same or distinct conditions and whether genetic effects on glycemic traits differ across populations. We find limited evidence for pregnancy-specific genetic effects and show that GDM loci with stronger effects in GDM than T2DM overlap with T2DM and/or glycaemic traits outside pregnancy. Classification of these loci into five biologically informed categories further reveals distinct patterns of pleiotropy, pregnancy-dependent effect modification, and diagnostic heterogeneity.

## Results

### Discovery of maternal and fetal genetic associations in multi-ancestry meta-analyses

Our GWAS meta-analysis included data from up to thirty cohorts across European (EUR), East Asian (EAS), South Asian (SAS), admixed Africans (AFR), and admixed Hispanics (HIS) populations (Supplementary Data 1-3). We conducted both ancestry-specific and multi-ancestry meta-analyses of maternal and fetal GWAS for five traits: i) GDM diagnosis (N_maternal_=38,305 cases and 776,145 controls; N_fetal_=3,126 cases and 90,877 controls), ii) FG (N_maternal_=55,371; N_fetal_=15,855), iii) 1hG post-OGTT (N_maternal_=38,439; N_fetal_=9,365), iv) 2hG post-OGTT (N_maternal_=46,401; N_fetal_=15,046) and v) HbA1c (N_maternal_=9,724; N_fetal_=7,035).

**Figure 2:**
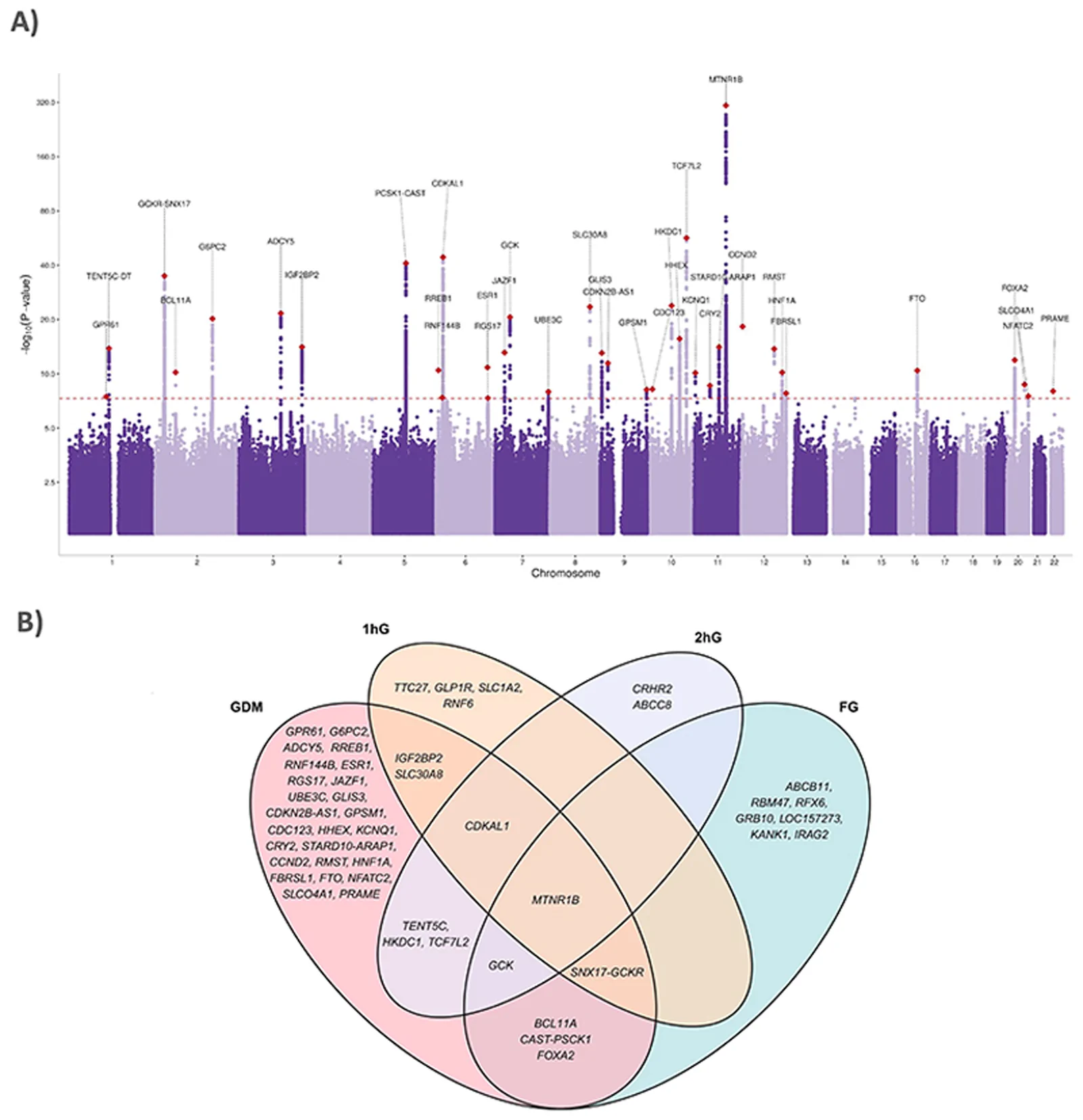
Summary of the associations for the maternal multi-ancestry GDM GWAS meta-analyses. (A) Manhattan plot for the maternal multi-ancestral GDM meta-analysis (38,305 cases and 776,145 controls), generated using MR-MEGA multi-ancestry meta-regression. Putative gene names labelled at genome-wide significant loci. The x-axis reflects chromosomal positions, and the y-axis reflects -log_10_(P) values for the two-tailed association test for each variant. (B) Venn diagram illustrating the genetic overlap between GDM and glycemic traits reaching genome-wide significance in the maternal multi-ancestry GWAS meta-analyses. Several loci showed evidence of shared genetic architecture between GDM and glycemic traits. *MTNR1B* variants were associated with all four traits. *CDKAL1* showed overlap with GDM, 1hG, and 2hG, while shared signals at *BCL11A, CAST-PCSK1*, and *FOXA2* (GDM and FG), as well as *IGF2BP2* and *SLC30A8* (GDM and 1hG) were also observed. Additional loci with trait overlap included *GCK* (GDM, FG, 2hG), *SNX17-GCKR* (GDM, FG, 1hG), as well as *TCF7L2, HKDC1* and *TENT5C* (GDM and 2hG). Sample sizes for the GWAS of glycemic traits were as follows: FG (N=55,371), 1hG (N=38,439), and 2hG (N=46,401). GDM; Gestational Diabetes Mellitus. FG; Fasting glucose. 1hG; 1-hour glucose after oral-glucose tolerance test. 2hG; 2-hour glucose after oral-glucose tolerance test.

In the multi-ancestry maternal GWAS meta-analyses, we identified 37 loci associated with GDM at genome-wide significance (P<5×10^-8^), including 7 novel GDM associations (Figure 2; Table 1; Supplementary Data 4, Manhattan plot: Supplementary Figure 1; Plots of the axes of genetic variation separating cohorts: Supplementary Figure 2; Locus zoom plots: Supplementary Figures 3-11; Previously reported GDM SNPs not replicated in our study: Supplementary Data 5; Genome-wide fine-mapping results for GDM in EUR: Supplementary Data 6). Findings from the multi-ancestry GDM analyses remained robust even after excluding small studies (<100 cases) (Supplementary Data 4).

We also detected several maternal loci associated with glycemic traits during pregnancy: 13 for FG, nine for 1hG, eight for 2hG, and four for HbA1c (Supplementary Data 7; Manhattan plots: Supplementary Figures 12-15; Plots of the axes of genetic variation separating cohorts: Supplementary Figures 16-19; Locus zoom plots: Supplementary Figures 20-27). Of these, five glycemic trait-locus associations were novel findings in pregnancy (*RBM47* and *IRAG2* in the FG analyses; *TTC27* in 1hG; *TCF7L2* and *ABCC8* in 2hG), although some are known to be associated with T2DM (Supplementary Data 7). Notably, several loci identified overlapped between GDM and glycemic traits during pregnancy (Figure 2). No additional loci were identified in the ancestry-specific meta-analysis (FUMA results for EUR and EAS GWAS are available in Supplementary Data 4, 7 and 8).

Fetal GWAS meta-analyses identified four loci associated with GDM and/or glycemic traits (Manhattan plots: Supplementary Figures 28-32; Plots of the axes of genetic variation separating cohorts: Supplementary Figures 33-37). However, DINGO analyses conditioning on maternal genotype suggested all signals were maternally driven, likely reflecting correlations with maternal genotypes rather than independent fetal effects (Supplementary Figures 38-45).

### Ancestry-specific variation in allelic effects

Our maternal multi-ancestry meta-analyses revealed significant heterogeneity in allelic effects across populations (P_ancestry_<0.05) (Supplementary Data 4 and 7), with several lead variants exhibiting discordant direction and/or magnitude of effect across ancestries (Supplementary Figures 46-55). This was captured by the meta-regression used in MR-MEGA, which partitions heterogeneity associated with population differences (represented by principal components (PCs)) and residual sources of variation (heterogeneity not captured by PCs). These differences were most evident between the two largest ancestry groups in our study - EAS and EUR - though effect estimates in the remaining populations in our analysis were accompanied by wide confidence intervals.

For GDM, notable differences in effect estimates between EUR and EAS were observed for variants in *IGF2BP2* (lead variant rs113851927), *CDKAL1* (rs9348441), *SLC30A8* (rs11558471), *SLCO4A1* (rs6122141) and *RMST* (rs77864822) (Supplementary Figure 46). Similar differences were observed for glycemic traits, including variants at *GCK* (rs730497), *LOC157273* (rs4841132), *IRAG2* (rs1683163), and *MTNR1B* (rs3847554) associated with maternal FG as well as *MTNR1B* (rs10765572) and *HKDC1* (rs5030937) detected in the maternal 1hG and 2hG analyses, respectively (Supplementary Figures 47-49). We further noticed a striking ancestry-specific pattern for *ABCB11* (rs2685806), where the effect on maternal FG was in opposite directions across EUR (β= -0.047, 95% CI: -0.058 to -0.035) and EAS (β= 0.066, 95% CI: 0.051 to 0.080) populations (Supplementary Figures 47 and 51).

### Residual heterogeneity and sensitivity analyses

We observed significant residual heterogeneity (P_residual_<0.05) for several variants associated with glycemic traits and GDM in the multi-ancestry maternal meta-analysis (Supplementary Data 4 and 7). This suggests that the PCs included as covariates in the meta-regression do not fully explain between-study variability, potentially due to differences in GDM screening/diagnostic criteria, OGTT load, the gestational age at which glycemic traits were measured, or other differences in the populations included.

To investigate potential sources of this variation, we conducted a sensitivity analysis, comparing cohorts based on the GDM ascertainment method to assess the contribution to residual variation in allelic effects detected in the GDM analyses. Out of 40 contributing datasets, 26 employed universal OGTT testing for GDM diagnosis (N_cases_=6,770, N_controls_=37,992), while 14 relied on risk-based testing or self-report (N_cases_=31,605, N_controls_=738,083). Among the 37 variants identified in the maternal multi-ancestry meta-analysis of GDM, only one lead variant (rs4665972 at the *GCKR-SNX17* locus) exhibited significant variability in the effect estimates between the testing approaches (P_sensitivity_=3.96×10^-4^) (Supplementary Data 9, Supplementary Figure 56). After accounting for the testing strategy, no significant residual heterogeneity was observed for this locus (P_residual_>0.05).

We then explored whether body mass index (BMI) could explain the opposite-direction effects observed across ancestries for maternal FG lead variant rs2685806 in *ABCB11* (Supplementary Data 9). However, sensitivity analysis revealed no evidence for BMI-correlated heterogeneity in the allelic effects at this locus (P=0.78).

### Heritability estimates and evidence of masking effects through gene-based testing

In EUR, observed SNP heritability estimates ranged from 0.014 ± 0.002 (liability SNP h2 = 0.108 ± 0.014) for GDM to 0.177 ± 0.085 for HbA1c, with intermediate values for FG (0.092 ± 0.025), 1hG (SNP h^2^ = 0.056 ± 0.058), and 2hG (SNP h^2^ = 0.076 ± 0.039) (Supplementary Data 10). EAS populations showed higher SNP heritability for GDM (observed SNP h^2^ = 0.053 ± 0.021; liability SNP h2 = 0.119 ± 0.047) and post-load glucose traits (1hG: SNP h^2^ = 0.144 ± 0.025; 2hG: SNP h^2^ = 0.112 ± 0.023) compared to EUR, while FG heritability was similar (SNP h^2^ = 0.075 ± 0.017) (Supplementary Data 10).

Given these modest SNP-based heritability estimates, we explored whether masking effects - where LD between causal variants with opposing or reinforcing effects attenuates marginal SNP associations, making them difficult to detect in single-variant GWAS - could partially explain this low heritability. Using mBAT-combo, a gene-based test designed to detect masking patterns, we identified several genes showing significant associations in mBAT but not in fastBAT after multiple testing corrections (α = 0.05/n genes), consistent with masking effects (Supplementary Figures 57-66; Supplementary Data 11 and 12). These masking effects were detected in both EUR and EAS analyses, reinforcing the notion that polygenic architecture and LD complexity contribute to the genetic basis of GDM and related traits.

### Genetic correlations with T2DM, glycemic traits and pregnancy complications

**Figure 3:**
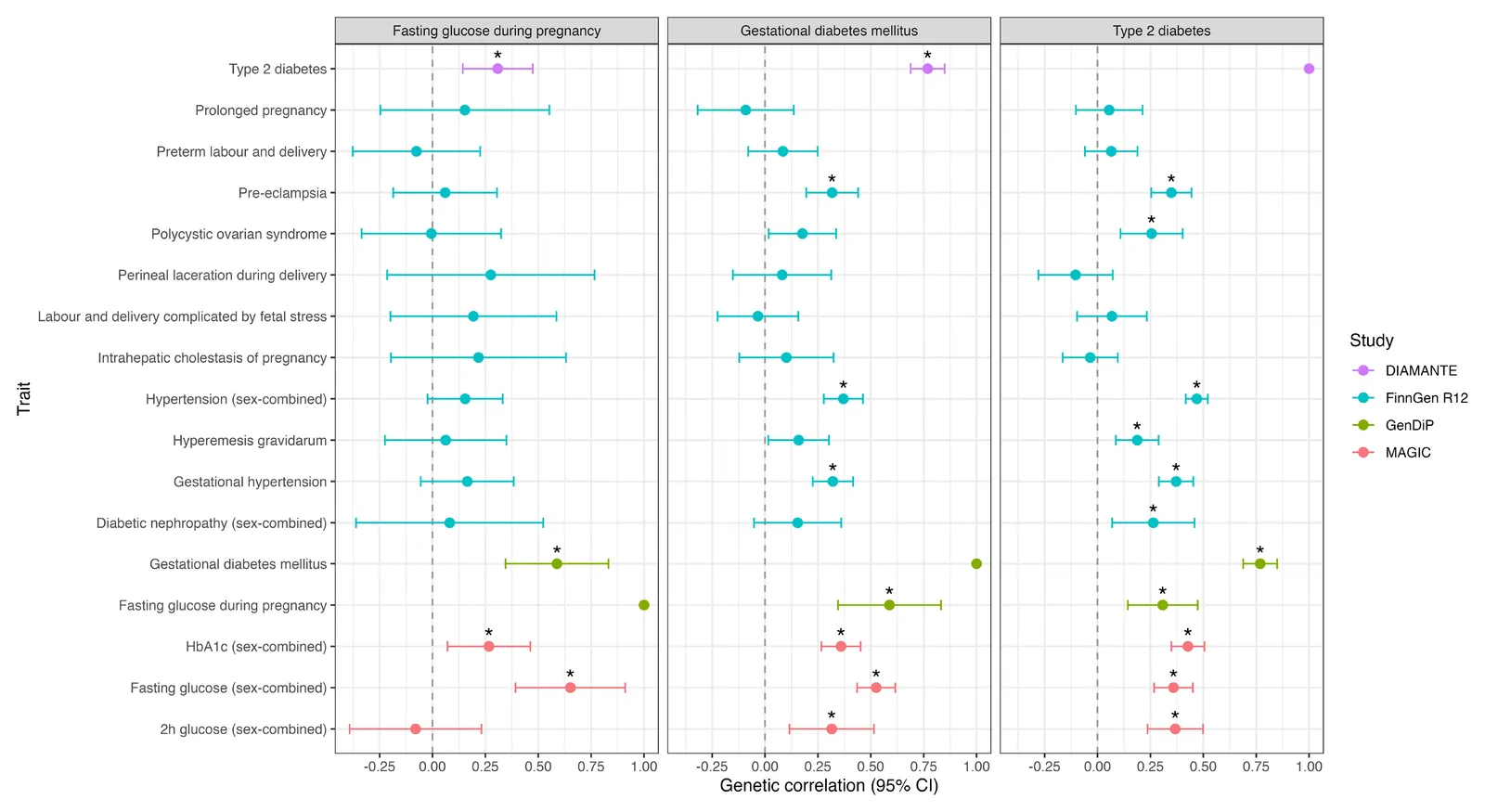
Genetic correlation analyses in Europeans. Panels display trait-specific genetic correlations for fasting glucose during pregnancy, GDM and T2DM computed using LD score regression. Each panel shows correlations with: (i) T2DM (purple; DIAMANTE consortium^70^), (ii) hypertension, adverse pregnancy outcomes and women’s reproductive health (blue; FinnGen R12^71^), (iii) pregnancy-specific glycemic traits (green; from our European GWAS meta-analyses), and (iv) glycemic trait measures outside of pregnancy (red; MAGIC consortium^46^). Genetic correlation estimates (rG) are presented as forest plots with point estimates and 95% confidence intervals (CI) as error bars, where the centre represents the estimated rG and error bars denote the 95% CI. Statistical significance was assessed using two-sided tests, with false discovery rate (FDR) correction applied across all tested traits. Asterisks indicate statistically significant associations after FDR < 0.05. The vertical dashed line at rG = 0 represents no genetic correlation. Sample sizes vary across traits and cohorts and are provided in Supplementary Data 13. GDM; Gestational Diabetes Mellitus. T2DM, Type 2 Diabetes Mellitus

To understand the shared genetic architecture between glycemic traits during and outside of pregnancy and assess the genetic overlap between these traits in pregnancy and pregnancy complications, we performed genetic correlation analyses. Our analyses revealed strong genetic correlations between diabetes-related traits in EUR (Figure 3; Supplementary Data 13; Supplementary Figure 67), with GDM and T2DM showing high genetic correlation (rG = 0.77, 95% CI = 0.69-0.85). GDM showed significant positive genetic correlations with most glycemic traits measured both during and outside of pregnancy, with rG ranging from 0.32 to 0.87. Notably, pregnancy measures of glycemic traits demonstrated moderate to strong genetic correlations with their counterparts outside of pregnancy. These patterns were similar, though less precise in EAS, with wider confidence intervals (Supplementary Figure 68). In EUR, GDM also showed positive genetic correlations with adverse pregnancy outcomes, including gestational hypertension (rG = 0.32, 95% CI = 0.23-0.42) and pre-eclampsia (rG = 0.32, 95% CI = 0.19-0.44) (Figure 3).

### Assessing the concordance in genetic effects between glycemic traits during and outside of pregnancy

To assess the degree of heterogeneity in genetic effects, we applied SCOUTJOY to compare GDM and T2DM and glycemic traits measured during versus outside of pregnancy. Genetic effects between GDM and T2DM were positively correlated in both EUR and EAS, using either sex-combined or female-only T2DM GWAS data (Supplementary Figures 69-72; Supplementary Data 14). Despite this overall concordance, up to 16 variants exhibited significant heterogeneity in effect sizes and were flagged as outliers, suggesting that pregnancy status may modulate their effects.

We also observed significant positive relationships between during and outside pregnancy for FG and 2hG traits, although the strength of associations with 2hG was smaller in magnitude (Supplementary Figure 73; Supplementary Data 14). Similarly to the GDM analysis, we detected outlier SNPs: four (EUR) and two (EAS) in FG analyses, and two (EUR) and one (EAS) in 2hG analyses.

### Classification of GDM-associated loci into variants with stronger effects in GDM or T2DM

To characterise the heterogeneity in genetic effects observed between GDM and T2DM in the SCOUTJOY analyses, we classified each GDM-associated lead variant based on whether its genetic effect was stronger for GDM or T2DM in both EAS and EUR populations. The classifications were largely consistent across ancestries; however, several variants could not be classified in the EAS analyses due to imprecise effect estimates arising from smaller GWAS sample sizes (Supplementary Data 15; Supplementary Figure 74-75).

**Figure 4:**
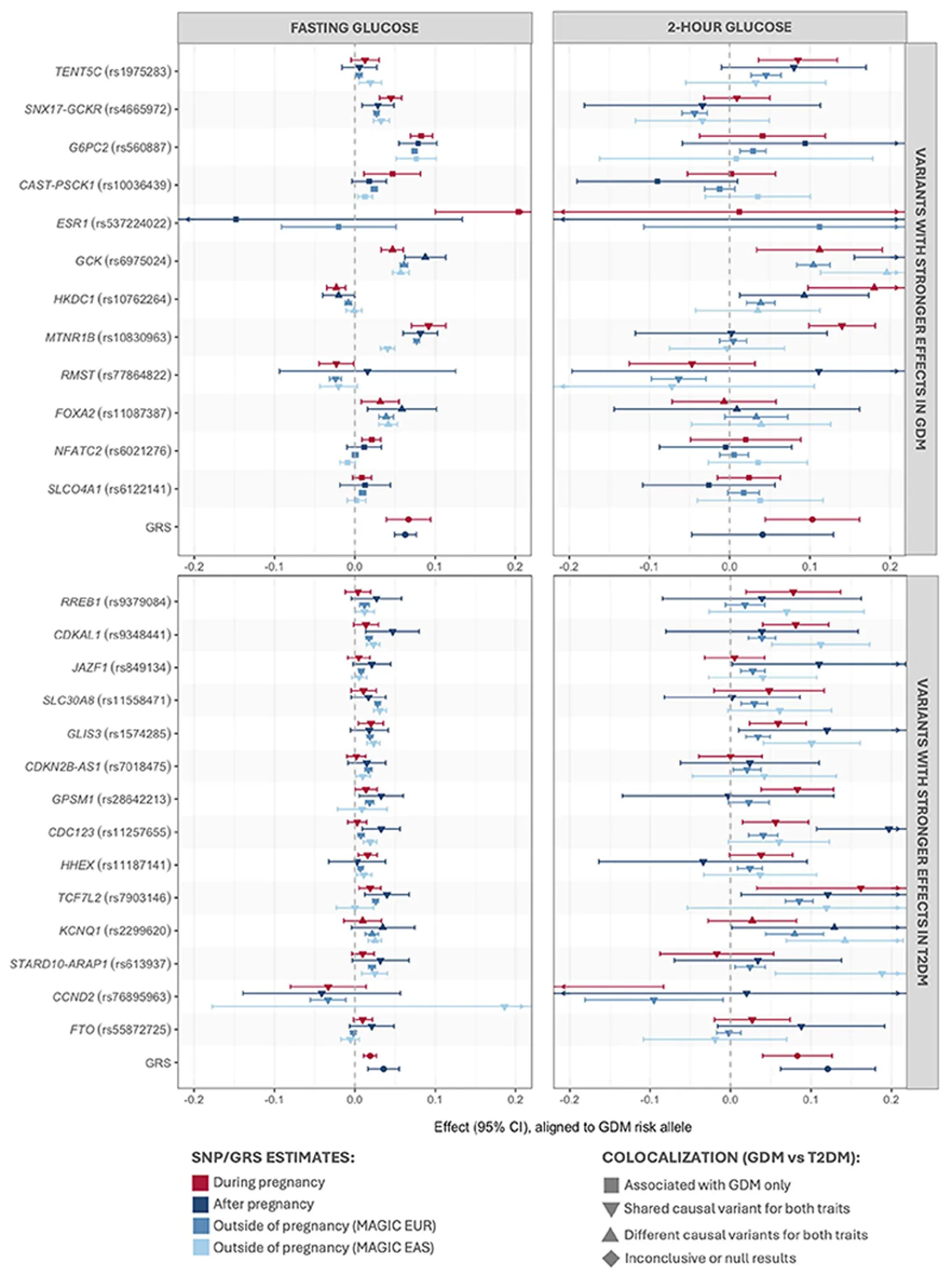
SNP and GRS associations with fasting and 2-hour glucose for GDM-associated loci. Figure shows the lead SNPs identified in the maternal multi-ancestry GDM GWAS meta-analyses using MR-MEGA, grouped by classification based on shared variant analyses in Europeans (EUR) and East Asians (EAS): variants with stronger effects on GDM, and variants with stronger effects on T2DM. For each SNP, we plot estimates for glucose: (i) during pregnancy (N=11,117 for FG and 9,850 for 2hG), (ii) after pregnancy in previously pregnant individuals (N=14,028 for FG and 4,307 for 2hG), (iii) outside of pregnancy in EUR individuals (N=200,622 for FG and 63,396 for 2hG; MAGIC consortium data^46^), and (iv) outside of pregnancy in EAS individuals (N=35,619 for FG and for 8,509 2hG; MAGIC consortium data^46^). Effect estimates are presented as beta coefficients with 95% confidence intervals (CI) as error bars and aligned to the GDM risk-increasing allele, with FG shown on the left and 2hG on the right panel. Estimates during pregnancy are shown in dark red, while those after or outside of pregnancy are in blue. Shapes denote colocalization with T2DM: squares, associated with GDM only; upward triangles, shared causal variant; downward triangles, distinct causal variants; diamonds, inconclusive or null results. Classifications reflect the overall pattern across populations (e.g. if colocalization suggested a shared causal variant in one ancestry and was inconclusive in the other, the locus was still classified as having a shared causal variant). For variants with stronger effects on GDM or T2DM (based on classification in EUR), corresponding genetic risk scores (GRS; 11 and 13 SNPs, respectively) are shown for pregnancy and post-pregnancy analyses (adjusted for principal components and maternal age). SNP and GRS effects during and after pregnancy were derived from our multi-ancestry cohort analyses. Arrows indicate truncated CIs. GDM; Gestational Diabetes Mellitus. T2DM; Type 2 Diabetes Mellitus. SNP; Single Nucleotide Polymorphism. EUR; Europeans. EAS; East Asians. GRS; Genetic Risk Score. FG; Fasting glucose. 2hG; 2-hour glucose after oral-glucose tolerance test.

Across both ancestries, 12 GDM lead variants showed evidence of stronger effects in GDM (lead variants in *TENT5C, SNX17-GCKR, G6PC2, CAST-PCSK1, ESR1, GCK, HKDC1*, *MTNR1B, RMST, FOXA2, NFATC2* and *SLCO4A1*), while 14 variants showed stronger effects in T2DM (Figure 4). Of the remaining variants, 10 could not be confidently classified in either population, and one (a variant in *RNF144B*) was not available in the EAS or EUR T2DM GWAS (Figure 4).

### Assessing evidence of shared causal variants for glycemic traits during and outside of pregnancy

We then conducted colocalization analyses to assess whether loci identified in the multi-ancestry analyses of GDM and glycemic traits during pregnancy share causal variants with T2DM and the corresponding glycemic traits outside of pregnancy.

Among the 37 genetic variants associated with GDM, colocalization analysis using data from EUR mothers revealed that six loci were associated only with GDM (*GPR61, G6PC2, CAST-PCSK1, ESR1, NFATC2*, and *SLCO4A1*), seven loci were associated with both GDM and T2DM but harboured distinct causal variants, 22 loci showed evidence of a shared causal variants with T2DM (Figure 4, Supplementary Data 16; Supplementary Figures 76-112). Colocalization results for GDM-T2DM were generally consistent between populations, although in EAS analyses, several loci showed inconclusive results or indicated that variants were not strongly associated with either trait.

Colocalization analyses of glycemic traits during vs outside of pregnancy identified nine loci with evidence of shared causal variants in at least one ancestry (Supplementary Data 16; Supplementary Figures 113-121). Of these, three FG loci (*GCKR-SNX17, GCK, MTNR1B*) colocalized with FG outside of pregnancy in both EUR and EAS datasets, while six loci colocalized with glycemic traits outside of pregnancy in only one ancestry: *RFX6* (FG) and *TCF7L2* (2hG) in EUR; *KANK1, FOXA2* (FG), *CDKAL1* (2hG), and *HBS1L* (HbA1c) in EAS. *ABCB11* (FG) and *GCK* (2hG) during and outside of pregnancy showed different causal variants in EUR and EAS, respectively, but with inconclusive results in the other population (Supplementary Figures 122-123). Five loci showed associations only during pregnancy: *ABCC8* (2hG) in both ancestries; *RBM47* (FG) in EUR analyses; and *IRAG2, TENT5C*, and *CRHR2* (2hG) in EAS analyses (Supplementary Figures 124-128). Colocalization results for four loci differed across populations: *CAST-PCSK1, GRB10* (FG), and *HKDC1* (2hG) shared causal variants in EUR but were only associated with the trait during pregnancy in EAS; *LOC157273* (FG) showed distinct causal variants in EUR and associations only in pregnancy in EAS (Supplementary Figures 129-132). Results for the remaining variants were inconclusive in both ancestries (Supplementary Figures 133-137).

### Associations of GDM-associated lead SNPs with glucose levels during and after pregnancy

To further explore whether GDM-associated variants reflect pregnancy-specific disruption of glucose regulation, we compared genetic risk score (GRS) associations with FG and 2hG levels measured during and after pregnancy. We analysed data from across six multi-ancestry cohorts, including women with glucose measurements during pregnancy (N=11,117 for FG and 9,850 for 2hG) and post-pregnancy (N=14,028 for FG and 4,307 for 2hG) (Supplementary Data 17). We constructed three maternal GRSs: a GRS including all 37 GDM-associated variants detected in the multi-ancestry analyses (All-GRS); a G-GRS including 11 variants that showed stronger effects in GDM than T2DM in EUR (Supplementary Data 15); and a T-GRS including 13 variants with stronger effects in T2DM than GDM in EUR (Supplementary Data 15).

The All-GRS and G-GRS showed similar associations with FG levels during pregnancy and post-pregnancy. In contrast, the T-GRS showed weaker associations with FG during pregnancy than post-pregnancy (Supplementary Figure 138; Supplementary Data 18-20). For 2hG, the All-GRS and T-GRS were strongly and consistently associated with levels during pregnancy and post-pregnancy. We also observed positive associations for the G-GRS with 2hG during pregnancy, and while the post-pregnancy GRS was seemingly weaker with 95% CI crossing the null, the CIs between the two overlapped (Supplementary Figure 139; Supplementary Data 18-20). Additionally, analyses of individual SNPs suggested greater effects on 2hG during pregnancy than post-pregnancy for the *MTNR1B* and *HKDC1* loci in class G, but power was limited (Figure 4; Supplementary Figure 140).

Overall, the GRS results remained consistent when adjusting for maternal age, restricting to EUR only, or only including women with glucose measurements both during and post-pregnancy and when weighting the GRSs by the T2DM effect estimates (Supplementary Data 19). Leave-one-out analyses suggested that no individual study influenced the between-study heterogeneity, except for the removal of i) HAPO (AFR), which strengthened the G-GRS association with 2hG during pregnancy and removed evidence of heterogeneity, and ii) HAPO (HIS) reduced the G-GRS association with 2hG post-pregnancy and removed evidence of heterogeneity (Supplementary Data 20).

## Discussion

Our multi-ancestry GWAS meta-analyses identified 37 independent genome-wide significant variants associated with GDM, including 7 novel GDM loci, as well as multiple genetic variants associated with glycemic traits during pregnancy. We found no evidence of fetal-specific genetic effects for any of the traits analysed. While most loci overlapped with T2DM and glycemic traits outside of pregnancy, a few loci showed stronger associations with GDM and context-dependent modulation of genetic effects in pregnancy.

A key strength of our study was the inclusion of diverse populations, which enabled the detection of ancestry-specific differences in allelic effects at several GDM- and glycemic trait-associated loci. A striking example is *ABCB11* (rs2685806; effect allele A), where the lead variant showed opposite directions of association in EUR (β=-0.047, SE= 0.006) and EAS (β=0.066, SE= 0.007) for FG during pregnancy - a pattern also observed for the same variant in FG outside of pregnancy after adjusting for body mass index (BMI), although not explicitly discussed by the authors^46^. Sensitivity analyses for FG incorporating mean cohort-level BMI as a meta-regression covariate found no evidence that BMI could account for this difference between ancestries, and imputation artifacts were unlikely given the use of distinct reference panels across cohorts. The consistency of ancestry-specific effects across cohorts also supported a genuine ancestry-related difference, perhaps reflecting differences in linkage disequilibrium (LD) structure. Consistent with this, colocalization in EUR suggested distinct causal variants for FG during versus outside pregnancy, whereas, although not definitive, EAS analysis suggested a shared causal variant (PP=0.68). Additionally, a look-up in Biobank Japan’s random glucose GWAS revealed that the EAS-specific lead variant rs74870851 (A) at *ABCB11* showed a direction of effect consistent with EUR, suggesting that rs2685806 may not be the true causal variant in EAS (the r^2^ between the variants is 0.809). Together, these results point to genuine ancestry-related differences at *ABCB11*, likely driven by variation in local LD structure, and reinforce the importance of multi-ancestry analyses to understand the genetics of complex traits.

We also observed that SNP heritability estimates for GDM and pregnancy-related glycemic traits remained modest across ancestries (e.g., EUR liability-scale SNP h^2^ for GDM = 0.108 ± 0.014). Although, differences in screening strategies and GDM diagnosis criteria across the cohorts could have contributed to this, another possible explanation is the presence of masking effects, where LD between causal variants with opposing or reinforcing effects attenuates marginal SNP associations^47^. Supporting this, our gene-based tests identified multiple genes in EAS and EUR showing evidence of masking effects and not detected in our standard GWAS, suggesting heritability may reside partly in loci with complex LD patterns or multiple weakly associated variants, which are poorly captured by traditional GWAS and LDSC regression.

To elucidate the genetic architecture of GDM and its relationship with T2DM, we examined GDM loci showing stronger effects on GDM than T2DM, integrating findings from several of our analyses (Tables 2 and 3). Unlike previous studies that variably labelled loci as “pregnancy-specific” often without robust comparison to non-pregnant contexts, our study classifies those loci with stronger effects on GDM into five categories reflecting more precise biological interpretations: (i) loci influencing diabetes and glucose metabolism exclusively during pregnancy, (ii) glycemic trait loci with no T2DM effect, (iii) glycemic trait loci with distinct causal variants affecting GDM and T2DM, (iv) loci sharing causal variants with T2DM but with effects modified by pregnancy status, and (v) glycemic trait loci sharing causal variants with T2DM, but with evidence of effect heterogeneity across GDM diagnostic approaches.

No loci met the criteria for pregnancy-specific effects (Category i), suggesting we have limited evidence of variants acting exclusively during pregnancy without influencing glycemia outside gestation. Consistent with this, our GRS including only variants with stronger effects on GDM showed similar associations with FG and 2hG during and after pregnancy, reinforcing limited, if existing, pregnancy-specific genetic effects.

However, we identified four GDM loci - *G6PC2, CAST-PCSK1, HKDC1*, and *FOXA2* - that were associated with glycemic traits outside of pregnancy but not with T2DM at genome-wide significance (Category ii). These loci also colocalized only with GDM and not with T2DM or harboured distinct causal variants from those affecting T2DM. Although individual SNP confidence intervals were wide, two loci showed suggestive trends toward stronger effects on glycemic traits during pregnancy: *CAST-PCSK1* in FG and *HKDC1* in 2hG. This pattern suggests that the effects of these variants may be amplified by the metabolic demands of pregnancy or may reflect differences in diagnostic thresholds between GDM and T2DM, contributing to their apparent pregnancy-specificity.

Additionally, at the *GCK* locus - a known glycemic trait locus showing genome-wide significant associations with both glycemic traits outside of pregnancy and T2DM - we observed evidence of distinct causal variants for GDM and T2DM (Category iii). Although variants in this locus are strongly associated with FG and 2hG outside of pregnancy^46^, our colocalization results suggest locus-level pleiotropy and potentially separate regulatory mechanisms for GDM and T2DM that are not necessarily pregnancy related.

We also observed evidence of pregnancy-dependent effect modification at *MTNR1B*, a well-established locus associated with glycemic traits outside of pregnancy (Category iv). While the causal variant is shared between GDM and T2DM, *MTNR1B* shows markedly stronger associations with 2hG during pregnancy. This pregnancy-amplified effect may be explained by its regulatory interaction with *GPR61* - a novel locus detected in our maternal multi-ancestry GDM meta-analysis - via receptor heteromerization^48^. Although *GPR61* remained unclassified in the shared variant analyses, it did not colocalize with T2DM or show genome-wide associations with glycemic traits outside of pregnancy. However, *GPR61* showed genome-wide significant associations in GWAS of anthropometric traits^49–51^, lipid biomarkers^52–54^, and metabolic syndrome^55^ - pointing to a broader metabolic role. Altogether, these findings support the existence of a gestation-specific melatonin-glucose signalling axis, where MTNR1B-GPR61 interaction potentially modulates the effect of *MTNR1B* during pregnancy.

Similar to *MTNR1B, GCKR-SNX17* is known to influence glycemic traits outside of gestation and was shown to share a causal variant between GDM and T2DM. Although individual SNP effects appeared to indicate a potentially stronger effect in FG during pregnancy, consistent with pregnancy-dependent effect modification, our sensitivity analyses revealed substantial heterogeneity in GDM effect estimates depending on glucose testing approach (i.e. universal vs risk-based) (Category v). This suggests that clinical practice variations could account for differences in effects for this locus and reinforces the importance of standardised testing and diagnostic criteria for GDM.

Several additional GDM loci that showed stronger effects on GDM than T2DM (*TENT5C, ESR1, RMST, NFATC2, SLCO4A1*) remained unclassified due to incomplete evidence. Overall, evidence suggests that GDM loci with stronger effects in diabetes during pregnancy represent a spectrum of genetic effects shaped by context, locus-level pleiotropy, and testing variability rather than uniformly “pregnancy-specific” mechanisms.

In addition to those loci, we identified several GDM loci with stronger effects on T2DM than GDM. Colocalization analyses indicated shared causal variants for GDM and T2DM at all but one locus - *KCNQ1*. Located in an imprinted region, *KCNQ1* harbours a likely distinct causal variant for GDM and T2DM and has been previously implicated in parent-of-origin effects, where maternally inherited risk alleles show stronger effects on diabetes-related traits^56^. Previous studies have demonstrated allele-origin-dependent regulation of *KCNQ1*, with placental DNA methylation in this locus liked to the regulation of maternal insulin sensitivity during pregnancy^57^. This interplay of opposing maternal and fetal regulatory effects may modulate its influence during pregnancy, potentially attenuating GDM effects and contributing to its stronger apparent effect on T2DM.

Our study has several limitations. First, while MR-MEGA enabled the integration of multi-ancestry genetic data, some signals (e.g. rs77090130) may reflect cohort-specific differences rather than true biological signals, as indicated by substantial heterogeneity and lack of significance in ancestry-specific analyses (Supplementary Note 5). While formal replication was not performed, many identified loci are well-established glycemic-trait signals and often showed associations across multiple ancestry groups, supporting the robustness of our findings. In addition, some previously reported GDM variants showed limited supporting evidence in our data, as outlined in Supplementary Data 5. Some of these reported associations, for example, could not be replicated due specific variants being present in limited number of cohorts in our main meta-analysis (MR-MEGA requires ≥5 cohorts), poorly imputed markers excluded during quality control, and incomplete availability of X-chromosome data. In some cases, previously reported variants were also inconsistently replicated in their original reports.

Another limitation of our study was the sample size imbalance - GDM cases predominantly EUR and glycemic traits largely powered by EAS cohorts - limited ancestry-specific discovery, stratified analyses and interpretation, and may also explain differences between EAS and EUR observed in follow-up analyses. This imbalance, together with small sample sizes for HbA1c and fetal GWASs, further constrained our power, particularly for HbA1c, where interpretation is additionally limited by pregnancy-related changes in red-cell turnover and the heritable component of glycation. Additionally, phenotypic heterogeneity across cohorts (e.g., differing GDM diagnostic criteria) may also have further increased heterogeneity and reduced power. Thirdly, our colocalization analyses assumed a single causal variant per locus, which may not hold in complex regions with multiple independent signals. Finally, minor sample overlap between large biobanks (e.g., FinnGen or UK Biobank) with smaller regional cohorts, could slightly inflate association signals, although this is expected to be minimal.

In conclusion, this multi-ancestry study provides the most comprehensive genomic analysis of GDM and pregnancy glycemic traits to date, identifying novel loci and uncovering both shared and distinct components with T2DM. Our findings demonstrate that GDM largely shares its genetic architecture with T2DM, with limited evidence for pregnancy-specific loci, while revealing ancestry-related differences in allelic effects and glycemic regulation during pregnancy. These findings underscore the importance of integrating diverse statistical approaches to refine locus interpretation and highlight the need for larger, ancestrally diverse studies of GDM and glycaemic traits during pregnancy to guide future precision medicine approaches.

## Methods

### Study cohorts

This was a multi-ancestry meta-analysis that included thirty cohorts of EUR, EAS, SAS, AFR, and HIS ancestries. A detailed overview of cohorts can be found in Supplementary Data 1. All studies were approved by the relevant institutional ethics review boards.

### Traits analysed

GWAS analyses were conducted for maternal genotype data (i.e. maternal genotypes and maternal GDM diagnosis/glucose measurement) and fetal genotype data (i.e. offspring genotypes and maternal GDM diagnosis/glucose measurement). When available, individual cohorts provided GWAS on (i) Fasting glucose (FG; mmol/l), (ii) 1-hour post-OGTT glucose (1hG; mmol/l), (iii) 2-hour post-OGTT glucose (2hG; mmol/l), (iv) HbA1c (%), and (v) GDM diagnosis (self-reported, ICD code or calculated from OGTT). All glycemic trait measures were obtained during pregnancy.

For the case-control GWAS both pregnancy cohorts and larger biobanks relying on ICD codes or self-reported diagnosis were included, therefore for some cohorts, controls might include both parous and non-parous women. Cohort contributions, phenotype definitions and individual cohort covariate adjustments can be found in Supplementary Data 1-3.

### Individual cohort analyses

Individuals were genotyped in each participating study/cohort, and stringent quality control measures were applied to both samples and SNPs. Sample data were pre-phased and subsequently imputed using reference panels appropriate to each cohort’s ancestry and design. Associations between genetic variants and both glycaemic traits and GDM were assessed using additive models based on the minor allele dosage and employing a regression framework involving principal components and study-specific covariates (such as maternal age, assessment centre, etc). Studies addressed population structure concerns by either excluding related samples and incorporating PCs as additional covariates in the regression model or by including a random effect for a genetic relatedness matrix within a mixed model. Study-level quality control, pre-phasing, imputation, and GWAS analysis procedure (including covariate and analysis tools) can be found in Supplementary Data 3.

### Quality control of GWAS

Prior to the meta-analysis and subsequent downstream analyses, quality control was performed by the GenDiP analysts, excluding SNPs exhibiting poor imputation quality (R^2^<0.3 or info score<0.4), minor allele count <10 or a minor allele frequency (MAF) of less than 1%. If required, genetic data were transformed to align with the NCBI’s Genome Reference Consortium human build 38 (GRCh38) using SNPTracker^58^, providing a standardised reference for subsequent analyses. Further, for the multi-ancestry analyses, odds ratios (ORs) and 95% confidence intervals (CIs) were calculated from the beta coefficients and standard errors provided by the original GWAS summary statistics.

### Meta-analyses

Ancestry-specific meta-analyses were performed using METAL (v 2017)^59^, aggregating either maternal or fetal GWAS summary statistics using a fixed-effect approach with inverse-variance weights to obtain effect size estimates within each of the five broad ancestry groups.

The multi-ancestry fetal and maternal GWAS meta-analyses were performed using MR-MEGA (v 0.2)^60^. Three PCs that defined axes of genetic variation were derived and employed as covariates, distinguishing GWAS within the five ancestry groups (i.e. AFR, SAS, EAS, HIS, and EUR). As our maternal GDM meta-analysis included several small cohorts, we also performed a separate multi-ancestry GDM meta-analysis excluding studies with less than 100 cases to validate the robustness of our findings.

After performing the multi-ancestry and ancestry-specific meta-analyses, we removed variants that were not present in at least half of the total meta-analysis sample size and variants with a MAF of less than 1%. Manhattan plots illustrating multi-ancestry GWAS results were generated using topr R package (v 2.0.2)^61^. Novel GDM variants were defined as those not previously reported in the literature and/or located >1Mb away from any previously reported variant.

### Multi-ancestry lead variants

From our multi-ancestry meta-analysis, independent lead variants were identified by first filtering for variants reaching genome-wide significance (P < 5×10^-8^) and then sorting these variants by chromosome and genomic position. Variants were clustered into genomic loci using a 1-megabase (Mb) proximity threshold, and within each locus, we selected the variant with the smallest P-value as the lead variant. Locus zoom plots were generated for genomic regions surrounding lead variants using locuszoomr R package (v0.3.8) to visualise association signals and define locus boundaries^62^. Gene track information for Homo sapiens (GRCh38, Ensembl v113) was obtained through AnnotationHub R package (v3.14.0). In case no protein-coding genes were present within ±250 kb of the lead variant, available long non-coding RNAs and microRNAs were visualised instead. Putative genes were assigned to each genomic locus based on positional proximity to lead variants.

### Functional annotation of European and East Asian GWAS

Functional annotation was performed for both EAS and EUR meta-analysis results using FUMA v1.7.0^63^. Genome-wide significant (P-value < 5×10^-8^) variants were assigned to genomic loci based on the LD r^2^ threshold of less than 0.6. All independent variants (r^2^ < 0.1) within a locus were considered to be lead variants, thus a single locus may contain more than one lead signal. Genomic loci in close proximity (< 250 kb) were merged into a single locus. The LD structure was based on the 1000 Genomes Phase 3 reference panel of the corresponding population (EAS, EUR)^64^.

### Fine-mapping of the European GDM results

To identify potential causal variants for GDM, we performed genome-wide fine-mapping of the European-ancestry GDM meta-analysis results using the Genome-wide Complex Trait Bayesian (GCTB) software (v2.5.4) with the SBayesRC algorithm^65^. Fine-mapping was conducted to identify putative causal variants and estimate their contribution to trait variance while accounting for LD. The analysis used block-wise full LD matrices computed from approximately 13 million SNPs in a European-ancestry sample from the UK Biobank, along with a functional annotation file, both provided by GCTB. Posterior inclusion probabilities (PIPs) and 95% credible sets were estimated genome-wide, with credible sets defined using default posterior thresholds of PIP ≥ 0.9 and posterior enrichment probability (PEP) ≥ 0.7.

### Maternal and fetal conditional analyses using DINGO

Maternal and offspring genetic effects on GDM, FG, 1hG, and 2hG were further analysed using the Direct and INdirect effects analysis of Genetic lOci (DINGO) software^66^. DINGO employs (i) conditional maternal analyses (adjusted for fetal genotype), (ii) conditional fetal analyses (adjusted for maternal genotype). Effective sample overlap between offspring and maternal GWAS was calculated using bivariate linkage disequilibrium score (LDSC) regression^67^. As LDSC regression does not perform well with small samples, DINGO was only performed with the EUR GWAS meta-analyses, as both the maternal and fetal sample sizes were the largest when compared to the other ancestry groups; However, the EUR HbA1c GWAS were too small to be included. For GDM, a binary outcome, genetic effects were transformed to the liability scale using the linear approximation before implementing DINGO^68^.

### Sensitivity analyses

To characterise the impact of GDM diagnostic criteria on the heterogeneity of allelic effects, we conducted a sensitivity analysis using cohort-level summary statistics of maternal GDM from 40 datasets, corresponding to 28 cohorts. We performed a meta-regression analysis using MR-MEGA (v0.2)^60^, where we employed universal diagnostic testing status (binary variable, i.e. use of universal blood-based diagnostic test) as an additional covariate alongside three principal components to account for diversity between ancestry groups. The universal diagnostic testing status was determined according to the phenotype ascertainment provided by contributing studies (Supplementary Data 1). We then examined the extent of heterogeneity arising from phenotype definition among lead variants identified in the multi-ancestry meta-analysis of maternal GDM. A likelihood-ratio test with one degree of freedom was used to assess the goodness of the extended model over the baseline model. The level of significance was set at 0.05/37=1.35×10^-3^, taking into account the number of tested lead variants.

Additionally, we characterised the impact of BMI on rs2685806 (*ABCB11* locus), the lead variant from the maternal FG multi-ancestry meta-analysis exhibiting opposite-direction effects in EAS and EUR populations. We extended the baseline model containing three ancestry-specific PCs in MR-MEGA by adding the mean BMI reported for 29 cohorts. Similarly to the GDM diagnostic criteria sensitivity analysis, the effect of BMI was assessed via a one-degree-of-freedom likelihood-ratio test.

### Heritability and genetic correlations

The LDSC regression software^67^ (https://github.com/bulik/ldsc) implemented in the Complex-Traits Genetics Virtual Lab^69^ (CTG-VL, https://vl.genoma.io/) was used to estimate SNP heritability (h^2^) of GDM and glycemic traits during pregnancy in both the EUR and EAS populations. We then assessed pairwise genetic correlations (rG) of our traits with publicly available summary statistics for T2DM (female-only sample, DIAMANTE consortium for EUR^70^ and AGEN for EAS^71^), hypertension (sex-combined, FinnGen R12 for EUR^72^ and KoGES for EAS^73^), and glycemic traits outside of pregnancy^46^ (sex-combined, MAGIC). For the EUR analysis, we additionally looked at 8 complications of pregnancy and delivery, as well as diabetic nephropathy (sex-combined) and polycystic ovarian syndrome, using the FinnGen publicly available dataset^72^. The overview of traits is provided in Supplementary Data 13. Summary statistics were filtered to the common, well-imputed SNPs from the HapMap 3 reference panel, and variants from the HLA region were removed. Pre-computed 1000 Genomes Project EUR and EAS LD scores^64^ available on the CTG-VL platform were used. Statistical significance was corrected for a false discovery rate of 5% using p.adjust function in R (v4.4.2). Observed-scale SNP h^2^ of GDM and corresponding SE were transformed to the liability scale^73^, where EUR and EAS population prevalences were presumed to be 12.3% and 14.7%^1^.

### Gene-based test

Gene-based analyses were performed using mBAT-combo^47^ implemented in GCTA (v 1.94.3)^74^ to identify putative genes associated with GDM, FG, 1hG, and 2hG. mBAT-combo assesses gene-trait associations by combining mBAT and fastBAT approaches using the Cauchy combination method, enhancing the detection of gene-level associations even in the presence of masking effects (e.g. SNPs with the same direction of effect but in negative LD). These analyses were conducted separately for both EUR and EAS using population-specific LD reference panels from 1000G^64^ and against the recommended gene list (GENCODE v40). The default settings were used, with mBAT-combo assigning SNPs to genes if they fell within 50kb upstream or downstream of the gene boundaries and allowing an allele frequency difference of 0.2 between the GWAS summary and LD reference dataset. A genome-wide significant threshold was used to identify genes significantly associated with the traits analysed (α =0.05/n genes; α =2.66×10^-6^ for EUR; α =2.75×10^-6^ for EAS).

### Heterogeneity in genetic effect estimates across traits

Significant Cross-Trait Outliers and Trends in Joint York regression (SCOUTJOY) was employed to assess the relationship and compare the heterogeneity of genetic effects among glycaemic traits and diabetes-associated loci^23^. This method extends the techniques introduced in MR-PRESSO to address heterogeneity detection by considering sample overlap and estimation error in both GWAS datasets^75^. In this approach, estimators for the York regression with a fixed intercept were employed to estimate the slope of the average relationship of the variant’s effect sizes for two phenotypes. This is followed by a global heterogeneity test based on the overall goodness of fit of the York regression model to the observed distribution of effect size estimates (i.e. testing if the observed effect sizes across top hits follow a single uniform relationship). Outlier variants were detected if they presented larger than expected York regression residuals. SCOUTJOY was implemented in R (v0.0.5).

The lead SNPs from the multi-ancestry GWAS meta-analyses were used for the analyses comparing the heterogeneity in genetic effects between (i) GDM and T2DM (sex-combined sample^25^ and female-only sample^70,71^. unadjusted for BMI), (ii) FG during and outside of pregnancy (sex-combined)^46^, and (iii) 2hG values during and outside of pregnancy (sex-combined)^46^. These analyses were conducted in both EUR and EAS; utilising SNPs identified in the multi-ancestry meta-analysis but with ancestry-specific effect sizes and standard errors (obtained from our ancestry-specific meta-analyses). Lead SNPs not present in the GWAS summary statistics downloaded or in the meta-analyses of glycemic traits conducted were excluded from the analyses. If the SNPs were not available in the datasets, we attempted to identify proxies; however, some SNPs were not present in the 1000G reference panel used in LDproxy^76^, had no variants in high LD, or did not have a proxy that reached genome-wide significance in the multi-ancestry GWAS (and was therefore not considered a suitable variant). Intercepts of the univariate and bivariate LDSC regression analyses required for SCOUTJOY were calculated before the analyses.

### Shared variant analysis

To explore whether lead SNPs identified in the multi-ancestry GDM GWAS meta-analysis exhibit genetic effects more strongly associated with T2DM or GDM, we applied the linemodels R-package (v.03)^77^ to summary statistics from both the GDM GWAS (Multi-ancestry: 38,241 cases and 775,169 controls; EUR: 29,625 cases and 736,101 controls; EAS: 4,064 cases and 22,276) and the T2DM GWAS in females (EUR: 30,053 cases and 434,336 controls^70^; EAS: 27,370 cases and 135,055 controls^71^). These analyses were run separately using EAS or EUR-specific GWAS. Although lead SNPs from the multi-ancestry GDM analysis were initially selected, comparisons used ancestry-specific effect estimates and standard errors; GDM SNPs absent from the T2DM dataset were excluded. The analyses ultimately included 34 GDM lead SNPs and 73 T2DM lead SNPs in the EUR analyses and 32 GDM lead SNPs and 40 T2DM lead SNPs in the EAS analyses.

Using bivariate effect sizes, SNPs were classified into two groups based on line models fitted with an Expectation-Maximization (EM) algorithm as implemented in Elliot et al., 2024^23^, producing slope estimates of 1.747 for one group (designated class T) and 0.2497 for the other (designated class G) in EUR and 1.082 for class T and 0.1443 for class G in EAS. Scale parameters for both groups were set to 0.2, while correlation parameters that defined the permissible deviation from the line were set to 0.99. Membership probabilities for each SNP in the two classes were calculated by a Bayesian classifier, assuming equal prior probability for each class. Due to the non-overlapping samples in the two GWAS datasets, the correlation of effect size estimates was set to 0. Variants were assigned to class T or G based on the highest posterior probability; those with a posterior probability ≥0.95 were labelled as GDM or T2DM, while those below this threshold were categorised as unspecified.

### Colocalization analysis

We conducted genetic colocalization analyses using the coloc R package^78^ to determine whether there were shared causal variants between GDM and T2DM for each locus identified in the maternal multi-ancestry GDM GWAS meta-analysis. These analyses were restricted to the maternal meta-analysis from participants of EUR and EAS ancestry due to sample size and availability of matching ancestry summary statistics. Additionally, we performed these analyses assessing the presence of shared variants for continuous traits - specifically FG, 2hG, and HbA1c - during versus outside of pregnancy. Summary data for pregnancy-related traits were derived from our meta-analyses, while publicly available data from the DIAMANTE (T2DM^25^) and MAGIC (FG, 2hG, and HbA1c^46^) consortia were used for non-pregnancy glycemic traits.

For each analysis, we extracted summary data for the relevant traits for SNPs within a +/-500kb window from the multi-ancestry GWAS meta-analyses lead SNPs. While the same lead SNP was used to define the region across ancestries for consistency and comparability, the colocalization analyses themselves were performed using ancestry-specific summary statistics. As coloc assumes the two GWAS have populations of comparable LD, this ensures that differences in LD structure across populations were appropriately accounted for, and that the analysis considered all variants within the locus rather than relying solely on the lead SNP.

We assumed a prior probability that any SNP in the region is associated with trait 1 only (in this case, GDM) (p1) of 1 × 10^-4^, trait 2 only (in this case, T2DM) (p2) of 1 × 10^-4^, or both traits (p12) 1 × 10^-5^. Coloc computes all possible configurations of causal variants for each of two traits and uses a Bayesian approach to calculate support for each causal model (H_0_: no association; H_1_: association with trait 1 only; H_2_: association with trait 2 only; H_3_: association with both traits owing to distinct causal variants; H_4_: association with both traits owing to a shared causal variant). We consider a posterior probability of association (PPA) for H_4_ > 0.7 to provide strong evidence for colocalization and PPA for H_3_ > 0.5 as evidence against colocalization, PPA for H_1_ > 0.5 as evidence for association with the pregnancy trait only, and PPA for H_2_ > 0.5 as evidence for association with the non-pregnancy trait only. While some studies adopt a more conservative threshold (e.g., H4 > 0.8), this cutoff is heuristic and not universally defined. As such, we also examined full posterior distributions to support locus-level interpretation, acknowledging that posterior probabilities should be treated as continuous measures rather than strict binary thresholds.

### Associations of GDM-associated lead SNPs with glucose levels during and after pregnancy

To test whether GDM-associated variants exert pregnancy-specific or consistent effects on glucose regulation, we compared the associations of genetic risk scores (GRS) with FG and 2hG levels measured both during and after pregnancy. We analysed data from 10 datasets, corresponding to six cohorts (some including multiple ancestries): EFSOCH (EUR), Gen3G (EUR), FinnGeDi (EUR), ALSPAC (EUR), HAPO (EUR, AFR, EAS, SAS, HIS), and ELGH (SAS). We analysed a total of 11,117 women with FG and 9,850 with 2hG levels during pregnancy (measured at a mean of between 24-28.4 weeks of gestation, mean maternal age ranged from 25.7 to 31.2 years at pregnancy) and 14,028 women with FG and 4,307 women with 2hG levels outside of pregnancy, with mean maternal ages across cohorts ranging from 27.0 to 47.4 years (Supplementary Data 17). Women were included if they did not have a diagnosis of diabetes (type 1 or 2) prior to pregnancy.

We analysed individual SNP associations but also grouped SNPs into GRSs to increase power. GRSs were constructed based on the SNP classifications derived from the shared variant analyses in EUR (11 SNPs with stronger effects in GDM and 13 SNPs with stronger effects in T2DM), and an overall GRS composed of all GDM variants identified in this study (n=37 SNPs), which additionally incorporated the 13 unclassified SNPs (G-GRS, T-GRS, and All-GRS, respectively). An individual’s GRS was calculated as the sum of the genotype dosages for each GDM increasing risk allele, weighted by its corresponding beta value (effect size, log-odds of GDM) from the EUR GWAS, given in Supplementary Data 4.

Associations were first estimated separately within each cohort using appropriate linear regression models, adjusting for the first 4 or 5 PCs and any study-specific covariables such as genotyping batches. Results from individual cohorts were combined using an inverse-variance weighted random-effects meta-analysis. We additionally analysed associations of individual SNPs and performed sensitivity analyses (i) including only women with glucose measurements both during and after pregnancy, (ii) conducting a leave-one-out cohort meta-analysis to assess heterogeneity, (iii) performing the meta-analysis in women of EUR ancestry only, and (iv) weighting genetic scores by log-odds of T2DM (given in Supplementary Data 15).

## Supplementary Material

Supplementary data

## Figures and Tables

**Figure 1:**
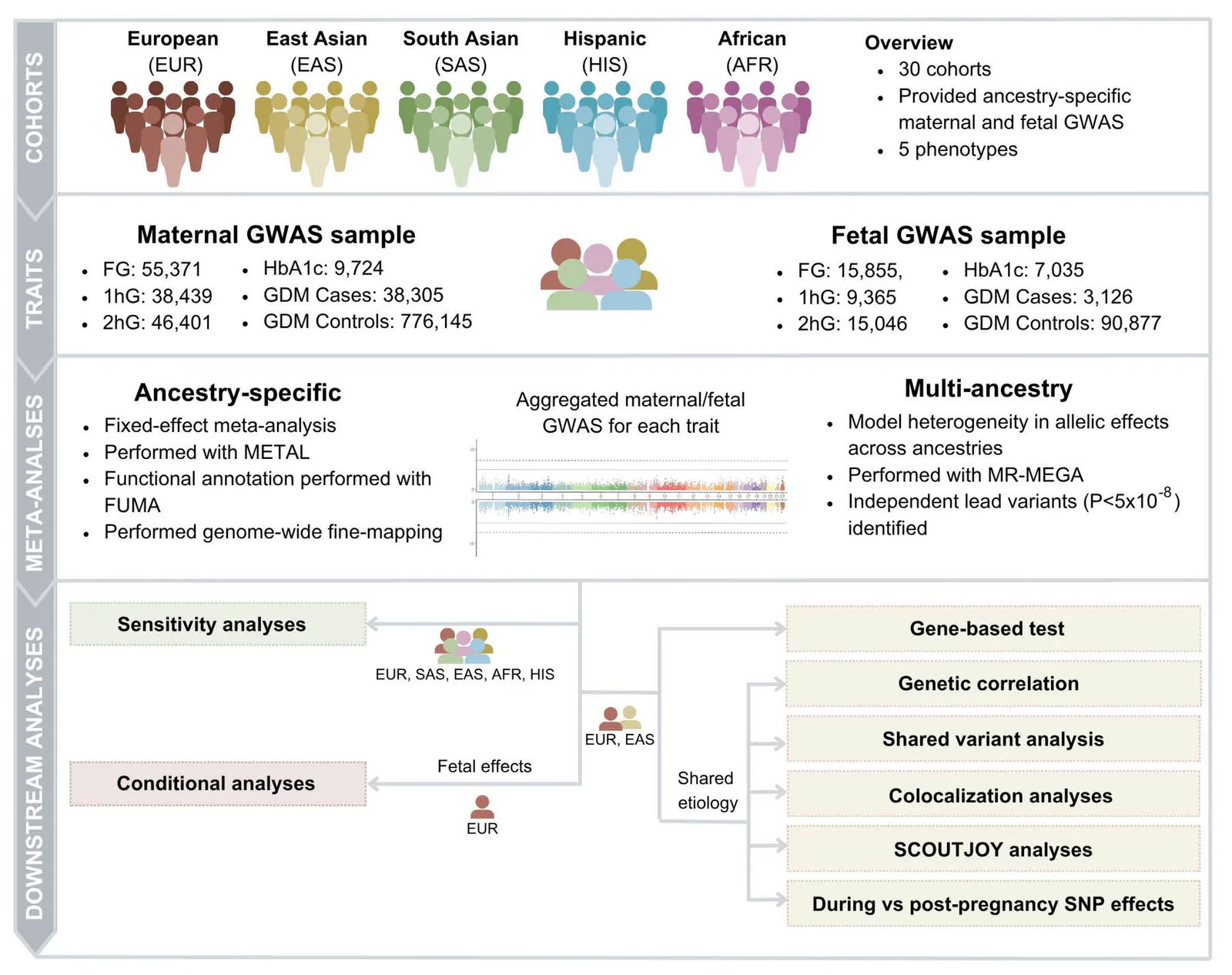
Study overview. Summary of the data resources, study sample and analyses conducted in this study. GDM; Gestational Diabetes Mellitus. FG; Fasting glucose. 1hG; 1-hour glucose after oral-glucose tolerance test (OGTT). 2hG; 2-hour glucose after OGTT. AFR: African. EAS; East Asian. EUR; European. SAS; South Asian. HIS; Hispanic.

**Table 1:** Summary of the novel independent lead variants identified in the multi-ancestry maternal meta-analysis of GDM. Associations between genetic variants and gestational diabetes mellitus (GDM) were estimated using MR-MEGA multi-ancestry meta-regression, with the association P-value corresponding to the two-sided test of association with GDM after accounting for heterogeneity correlated with ancestry. The ancestry P-values were obtained from a two-sided test of heterogeneity in effect sizes across ancestries, whereas the residual P-values were obtained from a two-sided test of residual heterogeneity assessing the degree of variation in the data that is not captured by the principal coordinates. GDM; Gestational Diabetes Mellitus. T2DM; Type 2 Diabetes Mellitus. AFR; African. EAS; East Asian. EUR; European. SAS; South Asian. HIS; Hispanic.

						Multi-ancestry meta-analysis				Significant in the ancestry-specific meta-analysis (P<0.05)
Lead variant	CHR	POS	Putative gene	EA	NEA	EAF	Association P-value	Ancestry P-value	Residual P-value	
rs41279738	1	109539929	*GPR61*	G	T	0.959	3.40×10^-8^	1.88×10^-2^	6.58×10^-1^	EUR
rs9379084	6	7231610	*RREB1*	G	A	0.893	3.51×10^-11^	1.64×10-2	7.37×10^-1^	EUR, SAS, AFR
rs142406759	6	18474036	*RNF144B*	G	GA	0.020	4.24×10^-8^	1.60×10^-6^	2.21×10^-1^	EUR, HIS
rs7767938	6	153046478	*RGS17*	C	T	0.743	4.65×10^-8^	1.33×10^-1^	9.00×10^-1^	EUR, EAS, SAS
rs28642213	9	136353630	*GPSM1*	G	A	0.257	6.81×10^-9^	4.25×10^-1^	8.53×10^-1^	EUR, SAS
rs11257655	10	12265895	*CDC123*	T	C	0.241	6.30×10^-9^	2.10×10^-3^	6.05×10^-1^	EUR, EAS, SAS
rs6122141	20	62642365	*SLCO4A1*	C	G	0.401	3.08×10^-8^	1.99×10^-3^	4.53×10^-1^	EUR, EAS

**Table 2:** Classification of GDM-associated variants based on underlying genetic architecture. GDM-associated variants with stronger effects in GDM than T2DM are classified into five categories based on multiple lines of evidence: (i) genome-wide association with T2DM (GWAS Catalog), (ii) genome-wide association with glycaemic traits outside pregnancy (GWAS Catalog), (iii) colocalisation analyses, (iv) SCOUTJOY outlier status, (v) individual SNP effects on FG or 2hG (from Figure 4) indicating relatively stronger effects on glycaemic traits during pregnancy, and (vi) evidence of heterogeneity in allelic effects related to GDM diagnostic testing (from MR-MEGA sensitivity analyses). GDM; Gestational Diabetes Mellitus. T2DM; Type 2 Diabetes Mellitus. SNP; Single Nucleotide Polymorphism. FG; Fasting glucose. 2hG; 2-hour glucose after oral-glucose tolerance test.

	Genome-wide associations		Evidence from our analyses			
Category	T2DM	Glycemic traits outside pregnancy	Colocalization	SCOUTJOY	SNP effect pattern	Testing heterogeneity
Pregnancy-specific loci	None	None	Distinct colocalization patterns	Flagged as an outlier	SNP estimates showing some suggestive trend where effects on glycemic traits (e.g. 2hG and FG) are greater during pregnancy	No
Glycaemic trait loci with no T2DM effect	None	Yes	Distinct colocalization patterns	Flagged as an outlier	SNP estimates showing some suggestive trend where effects on glycemic traits (e.g. 2hG and FG) are greater during pregnancy	No
Glycemic trait loci with distinct causal variants affecting GDM and T2DM	Yes	Yes	Distinct causal variants	Flagged as an outlier	SNP estimates showing some suggestive trend where effects on glycemic traits (e.g. 2hG and FG) are greater during pregnancy	No
Loci sharing causal variants with T2DM but with effects modified by pregnancy status	Yes	Yes	Shared causal variant	Flagged as an outlier	SNP estimates showing some suggestive trend where effects on glycemic traits (e.g. 2hG and FG) are greater during pregnancy	No
Glycemic trait loci sharing causal variants with T2DM, but with evidence of effect heterogeneity across testing approaches	Yes	Yes	Shared causal variant	Flagged as an outlier	Effect heterogeneity due to GDM diagnostic testing	Yes

**Table 3:** Classification of GDM-associated variants showing stronger effects in GDM than T2DM. This table summarises the classification of lead GDM-associated variants that exhibited stronger effects in GDM than T2DM based on the shared variant analyses results in at least one ancestry. For each locus, we report (i) the putative gene and lead SNP, (ii) summary of individual SNP effects on FG or 2hG (from Figure 4), (iii) whether the variant was flagged as an outlier in SCOUTJOY (in either EUR or EAS), and (iv) whether it showed heterogeneity in allelic effects attributable to GDM diagnostic testing (from MR-MEGA sensitivity analyses). We indicate whether each locus reached genome-wide significance for T2DM or glycemic traits outside pregnancy (in GWAS Catalog) and present colocalization results for (a) GDM vs T2DM and (b) glycemic traits during vs outside of pregnancy. Colocalization analyses were conducted separately in EUR and EAS using lead SNPs for FG or 2hG, which may differ from the GDM lead variant; loci not detected in glycemic trait analyses were not included in the colocalization results. GDM; Gestational Diabetes Mellitus. T2DM; Type 2 Diabetes Mellitus. FG; fasting glucose. 2hG; 2-hour glucose after oral-glucose tolerance test. EUR; Europeans. EAS; East Asians. SNP; Single Nucleotide Polymorphism

					Colocalization analyses	
Gene (rsID)	SNP effects	SCOUTJOY outlier	Testing heterogeneity	Genome-wide associations	GDM vs T2DM	Glycemic trait during vs outside of pregnancy
**Glycemic trait loci with no T2DM effect**						
*G6PC2* (rs560887)	No clear pattern	Yes	No	FG, HbA1c, HOMA-B	GDM-only (EUR); inconclusive (EAS)	-
*PCSK1* (rs10036439)	No clear pattern	Yes	No	Fasting proinsulin, FG	GDM-only (EUR); inconclusive (EAS)	Shared causal variants for FG (EUR); Only FG during pregnancy (EAS)
*HKDC1* (rs10762264)	↓ FG, ↑ 2hG (in pregnancy, suggestive)	Yes	No	HbA1c	Distinct casual variants (EUR)*; T2DM-only (EAS)**	Shared causal variants for 2hG (EUR); Only 2hG during pregnancy (EAS)
*FOXA2* (rs11087387)	No clear pattern	Yes	No	FG, HbA1c	Distinct casual variants (EUR)*; T2DM-only (EAS)**.	Only FG only outside of pregnancy (EUR); Shared causal variants for FG (EAS)
**Glycemic trait loci with distinct causal variants affecting GDM and T2DM**						
*GCK* (rs6975024)	↓ FG (in pregnancy, suggestive)	Yes	No	T2DM, random glucose, FG, HbA1c, 2hG, HOMA-B.	Distinct casual variants (EUR); inconclusive (EAS)	Shared causal variants for FG (EUR, EAS); Only 2hG outside of pregnancy (EUR); Distinct casual variants (EAS)
**Loci sharing causal variants with T2DM but with effects modified by pregnancy status**						
*MTNR1B* (rs10830963)	↑ 2hG in pregnancy	Yes	No	T2DM, HbA1c, FG, HOMA-B, random glucose.	Shared causal variants (EUR)	Shared causal variants for FG (EUR, EAS); Only 2hG during pregnancy (EUR); inconclusive (EAS)
**Glycemic trait loci sharing causal variants with T2DM, but with evidence of effect heterogeneity across testing approaches**						
*GCKR* (rs4665972)	↑ FG (in pregnancy, suggestive); 2hG (crosses null in pregnancy, suggestive)	Yes	Yes	T2DM, FG, random glucose, HbA1c, fasting insulin, 2hG.	Shared causal variants (EUR, EAS)	Shared causal variants for FG (EUR, EAS)

* Colocalization supports H_3_ (distinct causal variants) as both traits have regional signals, but the top SNPs and regional patterns differ for the two traits.

** EAS GDM GWAS yielded a weak regional signal due to small sample size, so colocalization with the larger T2DM GWAS supports association with T2DM only (H_2_), likely due to limited GDM power rather than absence of a true effect.

## Data Availability

The meta-analysis GWAS summary statistics generated in this study have been deposited in the University of Queensland eSpace repository and are publicly available under unrestricted access at: maternal multi-ancestry GWAS (https://espace.library.uq.edu.au/view/UQ:0aa6ba4), maternal ancestry-specific GWAS (EUR/EAS; https://espace.library.uq.edu.au/view/UQ:2e6c470), and DINGO analyses (https://espace.library.uq.edu.au/view/UQ:ed470a2). These meta-analysis summary statistics will also be deposited in the NHGRI-EBI GWAS Catalog (https://www.ebi.ac.uk/gwas/) upon publication. Fetal GWAS summary statistics and ancestry-specific GWAS for South Asian, Hispanic, and African populations have not been publicly deposited due to small sample sizes and limited contributing cohorts, which could potentially compromise participant privacy. However, DINGO results, which include effects from fetal EUR GWAS, are available in the deposited datasets (https://espace.library.uq.edu.au/view/UQ:ed470a2). These datasets can be shared on request subject to a data use agreement that includes provisions for maintaining participant confidentiality and restrictions on attempts to re-identify participants. Requests for access should be directed to the corresponding authors and will be reviewed with the cohorts contributing to the relevant GWAS within 30 business days. Access will be granted for research purposes only, subject to ethical approval at the requesting institution and execution of a data use agreement. GWAS summary results statistics for all cohorts were generated in the course of this study. Raw genotype data and GWAS summary statistics generated separately for each participating cohort in this study (for both maternal and fetal genotype data) prior to the meta-analysis are protected and not publicly available due to data privacy laws and data sharing agreements with individual cohorts. Access to these data is restricted and subject to approval through the respective cohort data access procedures. Information about individual cohorts can be found in Supplementary Note 1. Reference files used for GCTB genome-wide fine-mapping can be found on the GCTB website (https://gctbhub.cloud.edu.au/software/gctb/#Download). Reference files for gene-based tests are available on the GCTA website (https://yanglab.westlake.edu.cn/software/gcta/#mBAT-combo). Publicly available GWAS summary statistics from external studies used for genetic correlation analyses are listed in Supplementary Data 13. GWAS summary statistics for women’s reproductive outcomes and hypertension in Europeans can be accessed via the FinnGen website (https://www.finngen.fi/en/access_results, release r12). Hypertension GWAS in East Asians are available on Zenodo (https://zenodo.org/records/7042518). GWAS summary statistics for glycemic traits outside of pregnancy are available from the MAGIC consortium (https://magicinvestigators.org/). GWAS of T2DM in Europeans, European females, and East Asians can be accessed via the DIAGRAM consortium website (https://diagram-consortium.org/), and GWAS of T2DM in East Asian females are available from the AGEN consortium website (https://blog.nus.edu.sg/agen/summary-statistics/t2d-2020). All other data supporting the findings of this study are available within the article and its Supplementary Information files.
